# Digit ratio (2D:4D) and altruism: evidence from a large, multi-ethnic sample

**DOI:** 10.3389/fnbeh.2015.00041

**Published:** 2015-02-23

**Authors:** Matteo M. Galizzi, Jeroen Nieboer

**Affiliations:** Behavioural Research Lab, Department of Social Policy, London School of Economics and Political ScienceLondon, UK

**Keywords:** testosterone, digit ratio, social preferences, altruism, dictator game, C91, C92, D44, D81, D87

## Abstract

We look at the links between the *Digit Ratio*—the ratio of the length of the index finger to the length of the ring finger—for both right and left hands, and giving in a Dictator Game. Unlike previous studies with exclusively Caucasian subjects, we consider a large, ethnically diverse sample. Our main results are as follows. First, for Caucasian subjects we estimate a significant positive regression coefficient for the right hand digit ratio and a significant negative coefficient for its squared measure. These results replicate the findings of Brañas-Garza et al. (2013), who also observe an inverted U-shaped relationship for Caucasian subjects. Second, we are not able to find any significant association of the right hand digit ratio with giving in the Dictator Game for the other main ethnic groups in our sample, nor in the pooled sample. Third, we find no significant association between giving in the Dictator Game and the left hand digit ratio.

## Introduction

We report findings from a laboratory experiment with a large, multi-ethnic sample of subjects, where we investigate the link between subjects' *Digit Ratio* (DR) and giving in a Dictator Game (DG), a measure of *altruism*. The DR (also known as second-to-fourth digit ratio, or 2D:4D) is the length of the index finger divided by the length of the ring finger. The DR is associated with pre-natal exposure to sex hormones: it correlates negatively with testosterone and positively with estrogen exposure (Goy and McEwen, 1980; Manning, 2002; Lutchmaya et al., 2004; Malas et al., 2006; Hönekopp et al., 2007; Galis et al., 2010; Zheng and Cohn, 2011). Men have lower digit ratios than women, consistent with findings that the testosterone levels measured in amniotic fluid are higher for male fetuses (Gitau et al., 2005).

Pre-natal exposure to sex hormones occurs at a crucial stage for human brain development. The hormonal origins of variability in DR thus provide an explanation for the fact that DR correlates with social behaviors such as competitiveness, status seeking, and aggression toward others (Manning, 2002; Benderlioglu and Nelson, 2004; Bennett et al., 2010; Voracek et al., 2010; Hönekopp, 2011). In the economic domain, various studies have explored the links between DR and so-called social preferences. Social preferences are typically measured by observing actions in laboratory games with monetary earnings, such as the DG (Forsythe et al., 1994), the Ultimatum Game (UG; Guth et al., 1982), the Trust Game (TG; Berg et al., 1995) and the Public Good Game (PGG; Marwell and Ames, 1979). The different experimental games capture different aspects of social preferences, spanning from altruism (DG) and reciprocity (UG) to trust (TG) and cooperation (PGG). In all these experimental games, subjects are said to reveal social preferences if they take actions that diverge from standard Nash equilibrium predictions, notably the prediction that they will act to maximize their own earnings.

We focus on the DG and study the incidence of altruism in a game that involves allocating money between oneself and an anonymous stranger. A typical (and oft-replicated) result in DG experiments is that a substantial proportion of subjects allocate a positive amount to the stranger they have been paired with. Although the laboratory context in which these findings arise continues to be debated, they provide tentative evidence that many people are inclined to behave altruistically even when interacting anonymously without any prospect for reciprocation^1^.

Observing a statistically robust association between DR and behavior in social preferences games such as the DG would add to a growing body of evidence suggestive of a *hormonal* and *biological basis* for pro-social behavior (Kosfeld et al., 2005; Burnham, 2007; Zak et al., 2007; Baumgartner et al., 2008; Morhenn et al., 2008; Barraza and Zak, 2009; Cesarini et al., 2009; Coates et al., 2009; Millet and Dewitte, 2009; Zethraeus et al., 2009; Eisenegger et al., 2010, 2012; Sanchez-Pages and Turiegano, 2010). Although several studies have investigated these questions in laboratory experiments with social preferences games (e.g., Van den Bergh and Dewitte, 2006; Burnham, 2007; Millet and Dewitte, 2008; Van Honk et al., 2012; Chew et al., 2013), only four of these studies to date—summarized in Table 1—have directly explored the relationship between DR and behavior in social preferences games using real monetary incentives (Millet and Dewitte, 2006; Ronay and Galinsky, 2011; Buser, 2012; Brañas-Garza et al., 2013). Note that these four studies differ in terms of the procedure used to measure DR, the experimental games used to measure social preferences, and the participant pool. Of the two studies focusing on the DG, in particular, one finds a non-linear (inverted U-shaped) relationship between the DR and individual giving in the DG (Brañas-Garza et al., 2013), while the other finds a positive relationship (Buser, 2012). It should be noted, however, that the latter study uses a self-reported proxy, rather than a direct measure, for the DR.

**Table 1:** **Summary of studies on digit ratio and pro-social behavior in experimental games with real monetary incentives**.

	**Game**	**Measure**	**Hands**	**Sample and ethnicities**	***N*_M_, *N*_F_**	**Correlated?**
Brañas-Garza et al., 2013	DG	Scanned	Both	University of Granada students; Caucasian	95, 76	Yes, non-linear
Buser, 2012	DG, UG, TG, PGG	Self-reported	Both	University of Amsterdam students; Caucasian	69, 152	Yes, positive^*^ for DG, UG P.1, TG, and PGG; No for UG P.2
Millet and Dewitte, 2006	Modified PGG	Scanned	Right	University of Leuven undergraduate students; Not reported	27, 43	Yes, non-linear
Ronay and Galinsky, 2011	UG	Scanned	Right	Psychology students; Not reported	28, 20	Yes, positive^**^

*Game defines the type of experimental game: DG, refers to the Dictator Game; UG, to the Ultimatum Game; UG P.1, to the Ultimatum Game Player 1; UG P.2, to the Ultimatum Game Player 2; TG, to the Trust Game; PGG, to the Public Good Game. N_M_ and N_F_ refer to the number of male and female subjects, respectively*.

* *This study used a binary proxy for the DR and therefore the exact shape of the positive relationship is not known*.

** *This study reports a correlation only and therefore the exact shape of the positive relationship is not known*.

More important, the findings reported in Table 1 are either exclusively based on samples of Caucasian subjects or do not take ethnicity into account. This matters because ethnicity has been identified as an important source of between-subject variation in DR. Manning (2002), for example, reports that the variation of DR between ethnic groups, and even between Caucasians of different European origin, is larger than the variation between sexes within an ethnic group. This raises the question whether relationships between DR and behavior are sensitive to ethnicity—as Aycinena et al. (2014) report for the case of DR and risk taking.

To shed light on the issue of ethnicity in the empirical literature on DR and social preferences games, we conduct the first controlled laboratory study with an ethnically diverse subject sample. We purposely recruited from a multi-ethnic subject pool, resulting in a large sample with high proportions of Caucasian, Chinese and South-Asian subjects.

Our study focuses on altruism as measured by the DG, follows state-of-the-art procedures to obtain high-quality DR measures from hand scans (Neyse and Brañas-Garza, 2014), and reports data on *both* the DR for the right hand (henceforth RHDR—Right Hand Digit Ratio) and the left hand (henceforth LHDR)^2^. Our main findings are as follows. First, for Caucasian subjects we find a non-linear relationship between DG giving and RHDR: our estimates show a significant positive regression coefficient for the RHDR and a significant negative coefficient for its squared measure. This result is consistent with the findings by Brañas-Garza et al. (2013) who also found an inverse U-shaped relationship between DG giving and RHDR for Caucasian subjects. Second, we find no significant associations between the RHDR (either in level or in squared measures, jointly or separately) and individual giving in the DG, neither in our pooled, ethnically diverse, sample nor in any of the main non-Caucasian subsamples. Finally, we find no statistically significant association between the LHDR and giving in the DG.

## Methods

All experimental sessions were run at the Behavioural Research Lab at the London School of Economics and Political Science (LSE), London, between February and March 2014. The experimental protocol was approved by the LSE Research Ethics Committee. Subjects were recruited by e-mail from a mailing list of students that had previously registered for participation in experiments. There was no other eligibility or exclusion criterion to select subjects. In the email invitation, subjects were not informed about the exact nature of the experiment that would be conducted. They were only told that the experiment would last about an hour; they would receive £10 for their participation; and they would have the chance to get an extra payment related to some of the tasks. Subjects could sign up to any of five 1-hour sessions starting every hour between 10 am and 5 pm at every working day in the week.

A total of 746 subjects participated in our experimental sessions. Upon arrival, subjects were identified anonymously using an ID code assigned by the subject recruitment system (SONA), asked to read an informed consent form and to sign the latter if they agreed to participate in the experiment.

In the experimental session, subjects participated in a one-shot DG where they were (anonymously) matched with another subject in the same session. All subjects played the DG as *Player 1*, having to decide how to divide £10 between themselves and *Player 2*, a passive player who simply receives his share of the £10 as allocated by Player 1. Each participant was actually paid the amount of money they earned as Player 1 in the DG, in cash at the end of the experiment ^3^. Under standard assumptions, the Nash equilibrium of the DG is Player 1 allocating £10 to herself and 0 to Player 2. Any positive amount allocated to Player 2 can thus be interpreted as an expression of altruism. The DG was computerized and was programmed and implemented using Z-Tree (Fischbacher, 2007). It was followed by a questionnaire to gather information on individual socio-demographic characteristics, including their ethnicity ^4^.

At the end of the session, subjects were led into a separate room where the experimenter had set up a computer with a high-resolution scanner (Canon LIDE 110). Subjects were asked to read and sign a further informed consent form, which explained that they would be asked to place both of their hands on a scanner to obtain the DR (see Supplementary Material). They were reminded that placing their hands on the scanner was completely voluntary and that the data would remain strictly anonymous and confidential. There was no indication that any of the subjects knew or suspected that we were interested in the relationship between the DR and behavior in the DG. After subjects gave their consent, we obtained the scan of both LHDR and RHDR for each subject. The scans were made at the highest possible resolution (300 DPI); subjects were asked to remove any rings from their fingers and to place both hands flat on the scanner. To get the best possible image, we followed the measurement procedure described in Neyse and Brañas-Garza (2014) as closely as possible.

A total of 638 subjects gave consent for their left and right hands to be scanned. Note that this figure is likely an underestimation of the overall compliance rate as we lost some observations due to a technical issue with the scanner. We were able to link the DG data with DR for 602 of these subjects. We thus focus our analysis on these 602 subjects (81% of the original sample)^5^.

After the experimental sessions were completed, we recruited two research assistants to provide us with independent measures of the length of the second and fourth finger of each hand^6^. We calculated the digit ratios from the finger length measures and checked the correlation between the DRs implied by the measurements from the two research assistants. These correlations (0.895 for left hand, 0.867 for right hand) suggest that measurement was highly accurate. To obtain a single measure of the DR for our analysis, we computed the average of the two research assistants' ratios.

## Results

### Summary statistics

Our sample consists of 602 student subjects. The sample consists predominantly of female students (412 subjects, 68.44% of the sample) and is highly ethnically diverse: 221 subjects described themselves as Chinese (36.71% of the sample), 201 as White Caucasian (33.38%), 81 as South Asian (13.45%), 26 as Black (4.32%) and 73 as “Other” (12.13%). Females are predominant also in each ethnic group, representing 67.16% of Caucasian, 74.07% of South Asian, 69.23% of Chinese, and 53.84% of Black subjects in our sample. Given the small number of Black subjects and the composite nature of the “Other” ethnicities in our sample, in what follows we will mainly focus on the differences between the Chinese, Caucasian and South Asian groups.

### Digit ratios

Table 2 summarizes the measures of the LHDR and RHDR, in aggregate, and by sex and ethnicity-specific subsamples.

**Table 2:**
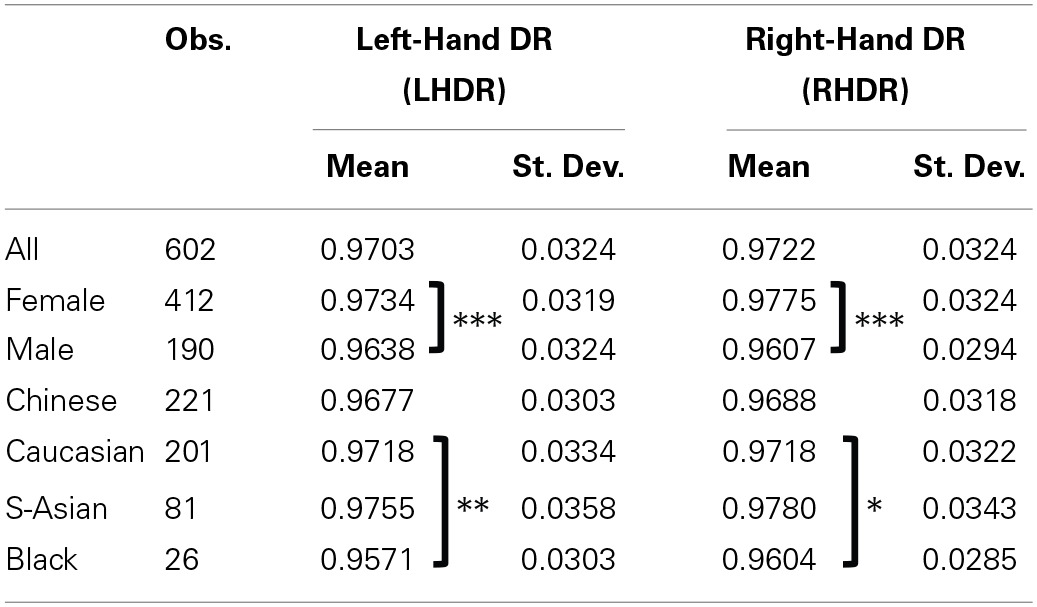
**Summary statistics for Left-Hand and Right-Hand Digit Ratios**.

Significant differences between sub-samples (two-tailed Mann-Whitney U tests) are shown as brackets. Stars indicate significance levels: ^*^p < 0.1, ^**^p < 0.05, ^***^p < 0.01.

Overall, both the LHDR and RHDR of male subjects are lower than those of female subjects. The average LHDR is 0.9638 (*SD* = 0.0324) for male subjects and 0.9734 (*SD* = 0.0319) for female subjects; the averages for RHDR are 0.9607 (*SD* = 0.0294) and 0.9775, (*SD* = 0.0324), respectively. Both differences are strongly statistically significant (two-sided Mann-Whitney U tests yield *p* = 0.0003 and *p* = 0.0000, respectively).

Whilst DR differences between sexes are strongly statistically significant in our sample, differences between ethnicities are not as clear cut. In general, the mean LHDR is 0.9677 (*SD* = 0.0303) for Chinese subjects, 0.9718 (*SD* = 0.0334) for White subjects, 0.9755 (*SD* = 0.0358) for South Asians and 0.9571 (*SD* = 0.0303) for Black subjects. Only the LHDR for Black subjects is statistically different from the LHDR of Caucasian subjects (two-tailed Mann-Whitney *U*-test, *p* = 0.0304). The LHDR for South Asian subjects is significantly different from the LHDR of Chinese subjects (two-tailed Mann-Whitney *U*-test, *p* = 0.0427) and of Black subjects (two-tailed Mann-Whitney *U*-test, *p* = 0.0153).

The mean RHDR is 0.9688 (*SD* = 0.0318) for Chinese subjects, 0.9718 (*SD* = 0.0322) for Caucasian subjects, 0.9780 (*SD* = 0.0343) for South Asians, and 0.9604 (*SD* = 0.0285) for Black subjects, with the latter being the only value (marginally) significantly different from the RHDR for Caucasian subjects (two-tailed Mann-Whitney *U*-test, *p* = 0.0652). Also the RHDR for South Asian subjects is significantly different from the RHDR of Chinese subjects (two-tailed Mann-Whitney *U*-test, *p* = 0.0329) and of Black subjects (two-tailed Mann-Whitney *U*-test, *p* = 0.0153). In general, the measures for RHDR and LHDR obtained for Caucasian subjects in our sample are broadly consistent with findings of previous studies using large samples of Caucasian subjects (e.g., Bosch-Domènech et al., 2014).

### DG giving

**Figure 1:**
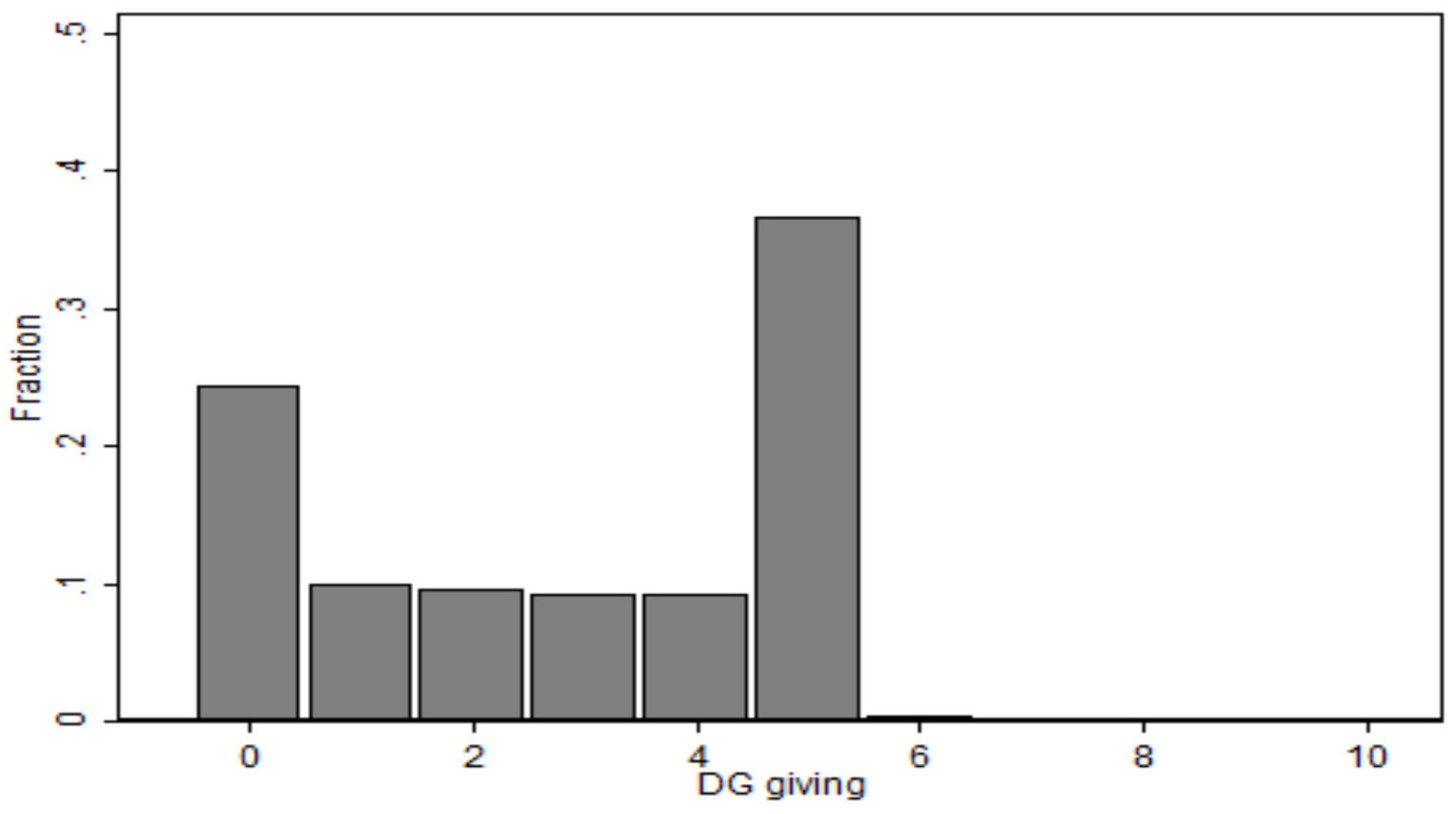
**Histogram of individual giving in the Dictator Game**.

Figure 1 and Table 3 summarize giving in the DG. As shown in Figure 1, the most common choices are to give nothing (24.42% of subjects) or the equal split (36.54%). The mean value for DG giving in our sample is 2.832 (*SD* = 2.101). Females in our sample were slightly more generous [mean giving of 2.919 (*SD* = 2.080) compared to 2.642 (*SD* = 2.137) for males] but this difference is not statistically significant^7^. We also find some significant differences between the giving behavior in the DG of different ethnicities. In particular, subjects describing themselves as of South-Asian ethnicity offered significantly more than Caucasian subjects (two-tailed Mann-Whitney *U*-test, *p* = 0.0074), and Chinese subjects (two-tailed Mann-Whitney *U*-test, *p* = 0.0020). Also, Black subjects offered significantly more than Chinese subjects (two-tailed Mann-Whitney *U*-test, *p* = 0.0020).

**Table 3:**
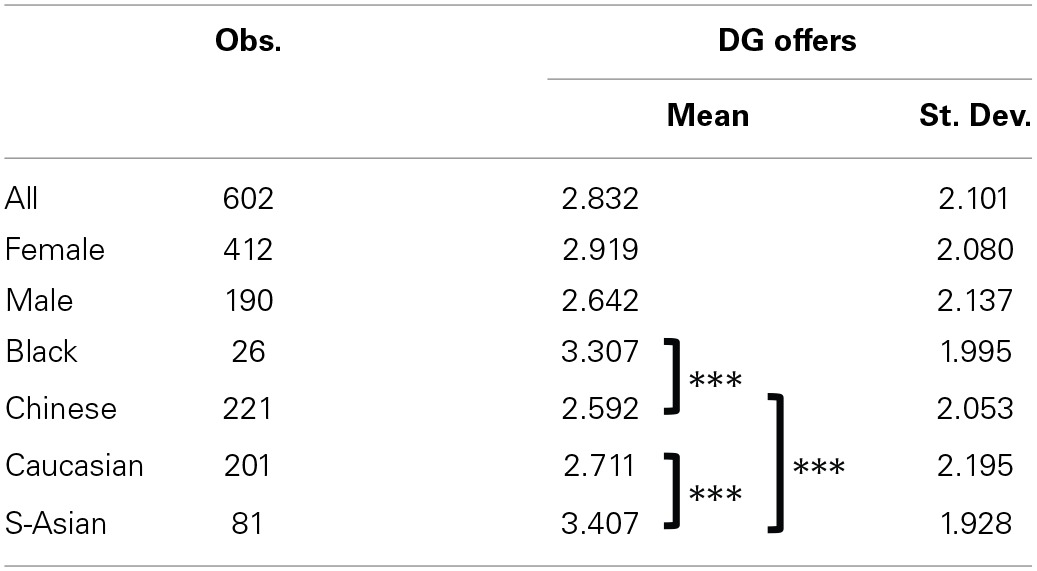
**Summary statistics for individual giving in the Dictator Game**.

Significant differences between sub-samples (two-tailed Mann-Whitney U test) are shown as brackets. Stars indicate significance levels: ^***^p < 0.01.

### Correlation analysis

We start by reporting pairwise correlations between the main variables of interest. We first note that, in our sample, LHDR and RHDR are strongly positively correlated (0.7212, *p* = 0.000). This is in line with previous literature (e.g., Bosch-Domènech et al., 2014) that typically reports 60–70% correlation between both hands' DR. Next, looking at the offers in the DG, we find negative but insignificant correlations between the offers in the DG and both the RHDR (−0.0023, *p* = 0.9542) and the LHDR (−0.0316, *p* = 0.4386). We obtain the same result when conducting the correlation analysis for sex- or ethnicity-specific sub-samples (not reported but available on request).

### Regression analysis

To explore the link between the DR and giving in the DG in more detail, we conduct a regression analysis that controls for subjects' sex and ethnicity. We model the relationship between DG giving and a set of explanatory variables using Ordinary Least Squares (OLS) regressions, adjusting the variance-covariance matrix for possible heteroskedasticity and serial correlation.

Our main analysis consists of two sets of regressions, repeated for each of the following samples: (i) all subjects; (ii) Caucasian subjects; (iii) Chinese subjects; (iv) South-Asian subjects. The first set of regressions estimates a linear relationship between DR on DG giving, controlling for respondents' sex. The second set of regressions adds the squared value of the DR into the regressions. Finally, we conducted multiple regression analysis to test for differences between sex and ethnicity sub-samples with respect to DG giving, and to account for gender-DR interaction terms^8^.

The regression results for RHDR (LHDR) are reported in Tables 4, 5. First, we note that the DR (RHDR or LHDR) on its own, entering the regression equation as a linear term, does not correlate with DG giving. When we model the relationship between DR and DG giving as quadratic, we note that neither the DR nor its squared term is significant for the full sample. When we repeat the analysis for sub-samples of our three largest ethnic groups, however, we find that the RHDR and its squared term do significantly associate with DG giving for Caucasian subjects. We find no evidence of a similar relationship for Chinese and South-Asian subjects. We also find no evidence of a relationship, linear or quadratic, between LHDR and DG giving for any of the ethnicity sub-samples.

**Table 4:** **DG Giving and RHDR (OLS)**.

**DG Giving**	**All subjects**	**Caucasian**	**Chinese**	**South-Asian**	**All subjects**	**Caucasian**	**Chinese**	**South-Asian**
RHDR	−1.172	−1.069	−2.193	−5.175	103.2	631.2^***^	−178.8	104.2
	(2.679)	(4.424)	(4.631)	(6.681)	(115.5)	(201.2)	(178.7)	(223.3)
RHDR squared					−53.60	−325.9^***^	90.81	−55.78
					(59.40)	(103.6)	(91.92)	(114.8)
Female	0.297	0.495	0.229	0.493	0.300	0.487	0.217	0.497
	(0.188)	(0.349)	(0.297)	(0.496)	(0.188)	(0.345)	(0.297)	(0.493)
Constant	3.768	3.418	4.559	8.104	−46.94	−302.8^***^	90.33	−45.49
	(2.588)	(4.255)	(4.471)	(6.489)	(56.08)	(97.62)	(86.84)	(108.5)
Observations	602	201	221	81	602	201	221	81
R-squared	0.004	0.010	0.003	0.017	0.005	0.050	0.007	0.021

Standard errors in parentheses. Stars indicate significance levels:

*** *p < 0.01*.

**Table 5:** **DG Giving and LHDR (OLS)**.

**DG Giving**	**All subjects**	**Caucasian**	**Chinese**	**South-Asian**	**All subjects**	**Caucasian**	**Chinese**	**South-Asian**
LHDR	−2.646	−1.096	−6.830	1.750	−35.33	237.2	−21.36	−16.11
	(2.666)	(4.549)	(4.718)	(5.322)	(104.4)	(199.9)	(164.8)	(161.4)
LHDR squared					16.81	−122.4	7.457	9.193
					(53.73)	(102.9)	(84.20)	(83.25)
Female	0.303	0.484	0.253	0.414	0.305	0.489	0.254	0.414
	(0.187)	(0.343)	(0.292)	(0.499)	(0.187)	(0.344)	(0.293)	(0.503)
Constant	5.192	3.451	9.027	1.392	21.06	−112.4	16.09	10.06
	(2.577)	(4.397)	(4.559)	(5.194)	(50.70)	(96.91)	(80.61)	(78.17)
Observations	602	201	221	81	602	201	221	81
R−squared	0.005	0.010	0.012	0.010	0.005	0.016	0.012	0.010

*Standard errors in parentheses*.

The shape of the estimated quadratic relationship between RHDR and DG giving for the Caucasian sub-sample is concave or inverse U-shaped, as in Brañas-Garza et al. (2013). Furthermore, the maximum of the estimated parabola is (0.968) is similar to the estimated maxima reported by Brañas-Garza et al. (2013) (0.956 for men and 0.961 for women) for a sample of Caucasian subjects. Like theirs, our estimated maximum is close to the center of the DR distribution (Caucasian subjects only, mean RHDR = 0.972, median RHDR = 0.974)^9^.

As a robustness check for the estimated quadratic relationship, we also estimate two separate linear (OLS) regressions between RHDR and DG giving, restricted to the data points of Caucasian subjects with RHDRs below and above the parabolic maximum of 0.968, respectively. These regressions, reported in Table A4 of the Supplementary Material, show a positive linear relationship between RHDR and DG giving below the maximum and a negative linear relationship above the maximum^10^. These results provide further evidence that the concave or inverse U-shaped relationship we observe is not an artifact of the quadratic statistical model. Finally, note that all the results we report here remain qualitatively identical when the analysis is replicated for sex- or ethnicity-specific subsamples; when gender-DR interaction terms are introduced^11^; or when the regressions are re-run using stepwise hierarchical regressions, censored Tobit models, or standardized z-values for the digit ratios (not reported but available on request).

## Discussion

For a large, multi-ethnic subject sample (*n* = 602), we investigate the relationship between the digit ratio (DR) of both hands and giving in a dictator game (DG) with real monetary incentives. In our study of the association between these two measures, we find three main results.

First, for Caucasian subjects we estimate a significant positive regression coefficient for the Right-Hand Digit Ratio (RHDR) and a significant negative coefficient for its squared measure. This result is not consistent with the findings of Buser (2012), but it is consistent with the findings of Brañas-Garza et al. (2013), who report an inverse U-shaped relationship between DR and DG giving^12^. In addition, our results are also quantitatively very similar to those reported by Brañas-Garza et al. (2013)—the maxima of the estimated parabolas are very close. This close match contributes to a more general body of evidence suggesting that the effect of biological measures on economic behavior is often non-monotonic (see also McFadden, 2002; Sanders et al., 2002; Sapienza et al., 2009; Sanchez-Pages and Turiegano, 2010; Nye et al., 2012). The idea of economic behavior as a function of deviations from a biological average—in either direction—certainly is a fascinating prospect that deserves further theoretical and empirical attention.

Second, we are not able to find any significant relationship between the RHDR (either in level or in squared measures) and DG giving in our non-Caucasian sub-samples, notably the Chinese or the South Asian ethnic groups. This suggests caution in generalizing associations between biological measures and behavior for subjects of one particular ethnicity to the whole of mankind. Whether the differences we observe are down to different ethnicities' conception of the DG and its context, ingrained cultural or social norms, or ethnic differences between DR and its hormonal origins, cannot be addressed with the current experimental design, however.

Third, we find no statistically significant association between the Left-Hand Digit Ratio (LHDR) and DG giving. This is not consistent with the findings of Brañas-Garza et al. (2013), who do find a relationship between LHDR and DG giving, although less robust than for the RHDR. The discrepancy between the findings on LHDR is consistent with the hypothesis that the RHDR is more representative of pre-natal exposure to sex hormones than the LHDR (see the meta-analysis by Hönekopp and Watson, 2010).

A limitation of our study design is its use of subjects from an ethnically diverse, but socially homogeneous, sample: university students. It has been argued that university students are a peculiar and unrepresentative sub-sample of the population (Enis et al., 1972; Cunningham et al., 1974; Gächter et al., 2004; Carpenter et al., 2008)^13^. How students attribute meaning to actions and outcomes in the DG may thus differ from the general population. Additionally, DG giving is only one way of operationalizing the measurement of social preferences. Social preferences can be measured using a broader set of experimental games such as the Ultimatum, the Trust, and the Public Good games. We welcome more research to systematically explore the association of biological and hormonal factors and social preferences.

### Conflict of interest statement

The authors declare that the research was conducted in the absence of any commercial or financial relationships that could be construed as a potential conflict of interest.

## Footnotes

^1^Much debate has focussed on whether the DG creates a context in which subjects feel compelled, by the design of the game, to act altruistically (e.g., Levitt and List, 2007; List, 2007; Bardsley, 2008).

^2^This in contrast to a substantial part of the DR literature that focuses exclusively on Right-Hand DR.

^3^Subjects were paid sequentially, in private, and were not informed of the outcomes of others.

^4^To get a standardized measure of ethnicity, we used the Self Defined Ethnicity (SDE) codes used by the United Kingdom's Home Office. Our subjects are familiar with this coding system, as it is widely used across the United Kingdom in official application forms for degree places, college accommodation, and jobs. None of the subjects refused to answer the ethnicity question in the questionnaire.

^5^To check for any selection bias of subjects with different characteristics into having their hands scanned, we compared the level of DG giving of subjects who did or did not have their hands scanned. For giving in the DG we cannot reject the null hypothesis that the mean of the two samples come from the same distribution (two-tailed Mann-Whitney *U*-test, *p* = 0.1597).

^6^The research assistants were told to take as much time as they needed to provide us with reliable measures. Both research assistants used Adobe Photoshop to measure the length of the fingers on the scans. They were instructed by the same experimenter to follow the procedures described in Neyse and Brañas-Garza (2014). The assistants were also given a copy of this procedure, for reference. The two research assistants did not know or meet each other and worked independently at different times. Research assistants had no access to the details of the subjects' whose digits they were measuring.

^7^Previous experimental evidence suggests that women are generally more pro-socially oriented than men (Croson and Gneezy, 2009). Although our results do not allow us to reject the null hypothesis that women and men are equally generous, the difference between sexes in our sample is in the same direction.

^8^These regressions are reported in Tables A1–A6 of the Supplementary Material. See also footnote 13 for a summary of the main findings.

^9^We also repeat Brañas-Garza et al. (2013) regression analysis of DG giving and deviations from the sample median (we do not have a population median as our sample is the only set of representative observations we have) and find similar results. These results are reported in Table A3 of the Supplementary Material.

^10^This approach is described by Nelson and Simonsohn (2014). Similar results are obtained following the recommendation of Lind and Mehlum (2010) about checking whether the optimum of our estimated quadratic relationship is within a reasonable domain.

^11^These regressions are reported in Tables A1–A6 of the Supplementary Material. The set of regressions confirm the earlier-reported results that the difference between female and male giving is not significant in our sample, as well as confirming significantly higher giving by South-Asian subjects. Furthermore, the regressions confirm that the introduction in the regressions of explicit gender-DR interaction terms does not alter the main findings reported below. In particular, neither the DR (RHDR or LHDR), nor the gender-DR interaction term significantly correlate with giving in the DG. There is no relationship, linear or quadratic between the LHDR, and DG giving, when the LHDR is entered in the regression together with its interaction with gender. When the relationship between RHDR and DG is modeled as a quadratic one, both the RHDR and its squared term do significantly correlate with DG giving for Caucasian subjects, even when an interaction term between the RHDR and gender is also included. No evidence of similar relationship, however, is found for the Chinese or the South-Asian groups, nor for the full sample of subjects.

^12^Note that Buser (2012) uses self-reported binary measures of DR, whereas Brañas-Garza et al. (2013) use directly measured high-resolution scans for the DR. Brañas-Garza and Kovarik (2013) show that the difference in results is due to a difference in measurement precision, with the latter procedure clearly superior.

^13^See, however, Stoop (2014) for recent studies finding that students do not exhibit significantly different social preferences from other subjects.
