# Supplementary material for: Digit ratio (2D:4D) and altruism: evidence from a large, multi-ethnic sample
Source: Front Behav Neurosci. 2015 Feb 23;9:41. doi: 10.3389/fnbeh.2015.00041 (PMC4337370; doi:10.3389/fnbeh.2015.00041)
Supplement: Supplementary file 1 [file Presentation1.PDF]

Digit ratio and altruism: Evidence from a large, multi-ethnic sample

Matteo M. Galizzi, Jeroen Nieboer

Supplementary Materials

Subject consent form for Digit Ratio measurement

Please read this consent form carefully and ask as many questions as you like before you decide whether or not you want to participate in the next measurement. Before you leave the laboratory today, we are asking everyone to take a measure called the digit ratio. This ratio is calculated by combining the length of your 2nd and 4th finger, and it has been shown in various scientific studies to correlate with people's behaviour in the laboratory. The most efficient and reliable way of measuring the ratio is by scanning someone's hand on a flatbed scanner.

As with all responses during our experiments, we will collect your digit ratio completely anonymously. No-one, not even the researcher in charge of the study, will be able to link your digit ratio to your identity, name, and personal information. As such, we will not be able to share your digit ratio with anyone, including you.

There are no risks to you from this research and no foreseeable direct benefits. It is hoped that the research will benefit others (or science) who wish to understand behaviour and decisions. The researcher in charge of today's study has collected digit ratio data in the LSE Behavioural Research Lab before. The image data will only be used for calculating the digit ratios, and it will be stored on an encrypted hard drive with no access to any external networks, kept in a secure storage space which will only be accessible by the researchers directly involved in this project.

If you have any questions about anything, please ask them now and/or contact the researcher in charge of the study: [contact details provided]. If you agree to provide a digit ratio measure, please continue.

****

I have read and understand this consent form and I am willing to provide a digit ratio measure

_________________________	_____________________________	____________
Signature	Name (please print)	Date
