# Supplementary material for: Digit ratio (2D:4D) and altruism: evidence from a large, multi-ethnic sample
Source: Front Behav Neurosci. 2015 Feb 23;9:41. doi: 10.3389/fnbeh.2015.00041 (PMC4337370; doi:10.3389/fnbeh.2015.00041)
Supplement: Supplementary file 2 [file DataSheet1.DOCX]

**Appendix. Tables**

**Table A1**: DG giving and individual characteristics: all subjects (OLS)

| *DG* | *m1* | *m2* | *m3* |
| --- | --- | --- | --- |
| Female | 0.277 |  | 0.274 |
|  | (0.185) |  | (0.185) |
| Chinese |  | -0.118 | -0.124 |
|  |  | (0.207) | (0.185) |
| South Asian |  | 0.695*** | 0.677*** |
|  |  | (0.264) | (0.263) |
| Black |  | 0.596 | 0.632 |
|  |  | (0.415) | (0.418) |
| Other |  | 0.371 | 0.367 |
|  |  | (0.288) | (0.288) |
| Constant | 2.642*** | 2.711*** | 2.527*** |
|  | (0.154) | (0.155) | (0.203) |
| Observations | 602 | 602 | 602 |

Standard errors in parentheses. * p<.10, ** p<.05, *** p<.01

**Table A2**: DG giving, RHDR, and individual characteristics: all subjects (OLS)

| *DG* | *m1* | *m2* | *m4* | *m5* |
| --- | --- | --- | --- | --- |
| RHDR | -0.151 | -1.172 | -0.866 | -1.903 |
|  | (2.643) | (2.679) | (2.643) | (2.674) |
| Female |  | 0.297 |  | 0.305 |
|  |  | (0.188) |  | (0.187) |
| Chinese |  |  | -0.121 | -0.131 |
|  |  |  | (0.208) | (0.207) |
| South Asian |  |  | 0.701*** | 0.686*** |
|  |  |  | (0.263) | (0.263) |
| Black |  |  | 0.586 | 0.614 |
|  |  |  | (0.415) | (0.418) |
| Other |  |  | 0.377 | 0.381 |
|  |  |  | (0.288) | (0.289) |
| Constant | 2.979*** | 3.768*** | 3.554*** | 4.357*** |
|  | (2.573) | (2.588) | (2.578) | (2.588) |
| Observations | 602 | 602 | 602 | 602 |

Note: Standard errors in parentheses; * p<.10, ** p<.05, *** p<.01.

**Table A3**: DG giving, RHDR distance from median, sex: Caucasian subjects (OLS)

| *DG* | *m1* |
| --- | --- |
| RHDR distance from median | -24.510*** |
|  | (7.882) |
| Female | 0.381 |
|  | 0.322 |
| Constant | 3.152*** |
|  | (0.352) |
| Observations | 201 |

Note: Standard errors in parentheses; * p<.10, ** p<.05, *** p<.01.

**Table A4**: DG giving, RHDR, sex: Caucasian subjects restricted based on RHDR (OLS)

| *DG* | *RHDR<0.968* | *RHDR>0.968* |
| --- | --- | --- |
| RHDR | 28.126** | -24.853** |
|  | (12.234) | (10.508) |
| Female | 0.468 | 0.434 |
|  | (0.470) | (0.496) |
| Constant | -24.064** | 27.174** |
|  | (11.500) | (10.451) |
| Observations | 89 | 112 |

**Table A5**: DG Giving and RHDR (OLS), with gender interaction term.

| *DG Giving* | *All subjects* | *Caucasian* | *Chinese* | *South-Asian* | *All subjects* | *Caucasian* | *Chinese* | *South-Asian* |
| --- | --- | --- | --- | --- | --- | --- | --- | --- |
| RHDR | .0140 | 3.096 | -2.978 | -6.651 | 102.392 | 641.686*** | 187.906 | 102.635 |
|  | (5.125) | (8.312) | (8.372) | (14.93) | (116.31) | (203.06) | (181.976) | (224.31) |
| RHDR squared |  |  |  |  | 53.135 | -332.02*** | 96.342 | -55.693 |
|  |  |  |  |  | (60.184) | (105.227) | (94.417) | (114.204) |
| Female | 1.873 | 6.414 | -0.7651 | -1.340 | 0.488 | -1.476 | 2.289 | -1.285 |
|  | (5.825) | (9.496) | (9.706) | (16.210) | (5.894) | (9.648) | (9.825) | (16.095) |
| Female*RHDR | -1.632 | -6.134 | 1.033 | 1.888 | -0.194 | 2.035 | 9.825 | 1.836 |
|  | (6.017) | (9.835) | (10.021) | (16.689) | (6.089) | (10.00) | (10.145) | (16.573) |
| Constant | 2.628 | -0.572 | 5.311 | 9.532 | -46.64 | -307.29*** | 93.980 | -44.0169 |
|  | (4.943) | (7.976) | (8.087) | (14.487) | (56.246) | (98.017) | (87.789) | (110.496) |
| Observations | 602 | 201 | 221 | 81 | 602 | 201 | 221 | 81 |
| R-squared | 0.004 | 0.012 | 0.003 | 0.018 | 0.006 | 0.050 | 0.007 | 0.021 |

Note: Standard errors in parentheses; * p<.10, ** p<.05, *** p<.01.

**Table A6**: DG Giving and LHDR (OLS), with gender interaction term.

| *DG Giving* | *All subjects* | *Caucasian* | *Chinese* | *South-Asian* | *All subjects* | *Caucasian* | *Chinese* | *South-Asian* |
| --- | --- | --- | --- | --- | --- | --- | --- | --- |
| LHDR | -6.759 | 4.569 | -14.771 | 0.827 | -23.467 | 240.098 | 24.337 | -19.027 |
|  | (4.535) | (8.200) | (7.443) | (10.612) | (103.268) | (200.618) | (167.465) | (160.521) |
| LHDR squared |  |  |  |  | 8.642 | -121.067 | -20.304 | 10.1660 |
|  |  |  |  |  | (53.392) | (103.263) | (86.868) | (82.615) |
| Female | -5.557 | 8.373 | -10.960 | 0.798 | 5.459 | 0.489 | -11.596 | -0.928 |
|  | (5.413) | (9.537) | (9.224) | (11.965) | (0.187) | (0.344) | (9.883) | (12.070) |
| Female*LHDR | 6.061 | -8.150 | 11.624 | 1.246 | 5.918 | -7.972 | 12.280 | 1.3790 |
|  | (5.590) | (9.844) | (9.524) | (12.283) | (5.635) | (9.934) | (10.200) | (12.390) |
| Constant | 9.157* | -2.014 | 16.675 | 2.290 | 17.222 | -116.444 | -2.145 | 11.971 |
|  | (4.377) | (7.9122) | (7.187) | (10.335) | (49.967) | (97.472) | (80.791) | (78.17) |
| Observations | 602 | 201 | 221 | 81 | 602 | 201 | 221 | 81 |
| R-squared | 0.007 | 0.014 | 0.018 | 0.010 | 0.007 | 0.019 | 0.012 | 0.011 |

Note: Standard errors in parentheses; * p<.10, ** p<.05, *** p<.01.
